# Explanations for the high potency of HPV prophylactic vaccines

**DOI:** 10.1016/j.vaccine.2017.12.079

**Published:** 2018-08-06

**Authors:** John Schiller, Doug Lowy

**Affiliations:** Laboratory of Cellular Oncology, Center for Cancer Research, NCI, Bethesda, MD, USA

**Keywords:** Human papillomavirus, HPV, Prophylactic vaccine, Virus-like particle

## Abstract

HPV L1 virus-like particle (VLP) vaccines administered in a prime/boost series of three injections over six months have demonstrated remarkable prophylactic efficacy in clinical trials and effectiveness in national immunization programs with high rates of coverage. There is mounting evidence that the vaccines have similar efficacy and effectiveness even when administered in a single dose. The unexpected potency of one dose of these VLP vaccines may largely be attributed to structural features of the particles, which lead to the efficient generation of long-lived antigen-specific antibody-producing cells and unique features of the virus life cycle that make the HPV virions highly susceptible to antibody-mediated inhibition of infection.

## 1 Introduction

**Fig. 1:**
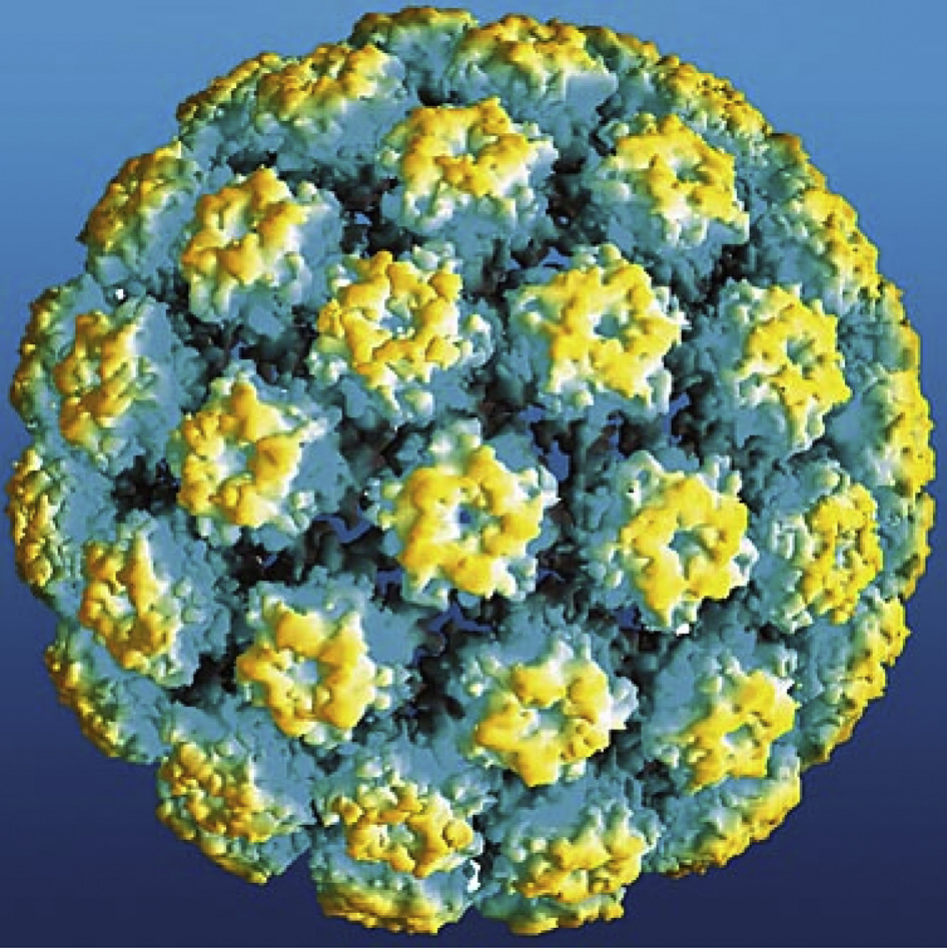
Atomic model of HPV16 L1 VLP, reproduced from [54].

The three commercial HPV prophylactic vaccines – Cervarix, Gardasil, and Gardasil-9 – are non-infectious subunit vaccines that contain virus-like particles (VLPs) of, respectively, HPV 16 and 18; HPV 6, 11, 16, and 18; and HPV 6, 11, 16, 18, 31, 33, 45, 52, and 58. The VLPs form by the self-assembly of 360 copies of the L1 major capsid protein of the virus (Fig. 1) [1]. Clinical trials that specified intramuscular injection of three vaccine doses over a six month period demonstrated high efficacy in preventing persistent incident infections and pre-malignant neoplasias induced by the HPV types targeted by the respective vaccines [2]. Vaccinees rarely tested positive for HPV infection at even a single time point, as measured by sensitive PCR assays, implying that the vaccines provide “sterilizing” immunity from initial infection in most cases. Most “breakthrough” infections in vaccinees appeared in the early months in the trials, suggesting that most of these infections represent emergence of infections preexisting at the time of vaccination, rather than new infections after vaccination [3].

There is also accumulating evidence for high effectiveness of Cervarix and Gardasil in national immunization programs [4], [5]. Post hoc analyses of three clinical trials, detailed in companion articles, have provided evidence that strong protection is induced in young women even after a single dose [6], [7], [8]. In addition, surveillance studies strongly suggest that a single dose can reduce infection and neoplastic disease incidence in national immunization programs, although, as discussed in another companion article, these studies are subject to confounding biases, including differential risk for preexisting infection in single dose recipients [9], [10].

The high degree of efficacy and effectiveness exhibited by the HPV vaccines, potentially even after a single dose, is exceptional for two reasons. First, no other vaccine has been successful developed against a microbe that is primarily sexually transmitted, despite considerable effort in the public and private sectors. Second, other licensed subunit vaccines are administered in a series of two or more prime/boost immunizations. It is therefore interesting to consider what factors may contribute to the unanticipated potency of the HPV vaccines. We believe that the two most important aspects are the ability of the vaccines to consistently induce high and durable titers of infection-inhibiting antibodies and an exceptional susceptibility of the virus to antibody-inhibition of infection in its target tissue. In this review, we discuss why antibodies are likely to be the prime mediators of protection, why the VLPs are exceptionally strong inducers of durable antibody responses, and why the virus life cycle makes it especially responsive to antibody-mediated inhibition. Together, these explanations provide a biologically plausible rationale for why the HPV VLPs may be the first subunit vaccine to exhibit long term effectiveness after a single dose.

## 2 Mechanisms of protection

Several lines of evidence support the conjectures that infection-inhibiting antibodies are the principal mediators of HPV vaccine-induced protection and that cell-mediated immune effector responses play, at best, a more limited role, although they are part of the immune response to the vaccine. First, as discussed in more detail below, high and durable serum titers of VLP antibodies are consistently generated by the vaccines, and these antibodies readily neutralize the virus in *in vitro* assays. Second, antibody-mediated neutralization, like protection in the trials, is type-restricted, with the limited cross-type protection observed in clinical trials largely mirroring the antibody-mediated cross-neutralization observed *in vitro* [11]. Third, protection can be passively transferred in serum drawn from vaccinated individuals to naïve individuals in animal challenge models [12], [13], [14]. Fourth, cell-mediated effectors generally function only after infection occurs, while sterilizing immunity was observed in the clinical trials. Fifth, the vaccines had no observable effect on established infections, although such effects would be expected if cell-mediated mechanisms were primarily responsible for protection [15], [16], [17]. In this context, it is important to note that L1 is primarily a nuclear protein that is not displayed on the surface of infected cells, which makes it unlikely that L1 antibodies can induce regression of established infections/lesions by antibody-dependent cytotoxicity. Therefore, the L1 antibodies are likely to function exclusively by preventing initial events during the infection process.

However, one observation that is difficult to reconcile with an antibody effector mechanism for protection are the findings from the Cervarix phase 3 trial that protection from high grade cervical precancers (CIN3) associated with non-vaccine types appears to be stronger than protection against incident infection by the same types [18], [19]. Studies of the impact of Cervarix in the Scottish immunization program support the high level of cross-protection for CIN3 observed in the clinical trial, in that the rates, irrespective of the HPV type, have decreased by more than 90% in young women who were vaccinated with Cervarix at age 13 and screened at age 20 [20]. How the vaccine could differentially induce cross-protection at the level of high grade disease is unclear.

One possible explanation for the differential protection at the level of CIN3 may be that the vaccine induces T cell responses to L1 that potentially could be cross-type protective (and there is some limited evidence to support this conjecture [21]), but that expression of L1 is normally too low in the basal epithelial cells, where productive infections are maintained (discussed below), for the infected cells to be targeted by cell-mediated responses. CIN3s are thought to arise mainly from high-risk HPV infection in a specific subset of cells in the cervical squamocolumnar junction that retain certain embryological characteristics [22]. It is possible that L1 is expressed at sufficient levels in these unusual cells to make them preferentially susceptible to type cross-protective T cell responses, thereby leading to preferential elimination of the infected cells destined to produce CIN3. Consistent with this possibility, VLP vaccination can induce regression of transplantable subcutaneous tumors that express very low levels of L1 in a mouse model [23]. The presence of an immunosuppressive microenvironment in established infections/neoplasia [24] may prevent these mechanisms from effectively functioning to induce lesion regression if the vaccines are administered in a therapeutic setting.

If antibodies are the primary mediators of protection, the question arises as to whether persistent levels of antibodies need to be maintained long term so they are present at the time of initial virus exposure or whether an anamnestic response after exposure, mediated by memory B cells, can protect from persistent infection and subsequent disease. There is precedence for the latter possibility. For example, individuals vaccinated with a hepatitis B virus (HBV) vaccine can become transiently infected, as evidenced by seroconversion for non-vaccine viral antigens, but never become symptomatic [25]. However, it is most likely that neutralizing antibodies need to be present at the time of exposure for the HPV vaccines to be most effective.

The female genital tract is generally considered to be a poor inducer of antibody responses, presumably in part to limit infertility that could result from the induction of anti-sperm antibodies [26]. In keeping with this idea, intravaginal delivery of 5 µg HPV16 VLPs, a relatively high dose, induced little if any antibody response in mice unless the tissue was chemically disrupted [27]. Although the virion antigen load that is transferred from an infected sexual partner is not well documented, it is likely to be relatively low, too low to readily induce an anamnestic response. Consistent with this conjecture, increases in VLP antibody titers, once they have stabilized after vaccination, are rare in sexually active women, although these women are fully able to mount a strong anamnestic response to an additional injected dose of the vaccine [28].

One could postulate that a breakthrough infection at a genital site with low propensity for carcinogenic progression, e.g. the vaginal wall, could induce a recall antibody response that would protect against successive rounds of auto-inoculation, which could otherwise lead to infection of the cervical transformation zone with high probability of progression. However, if this scenario occurred commonly, then vaccination of women with prevalent infection would be expected to have a reduced rate of progression to high grade precancer, but this type of protection was not observed in the clinical trials [15], [17].

Although 40% of vaccine recipients in the Gardasil trials were reported to become seronegative for HPV18 by four years post-vaccination, there was no evidence that Gardasil was less protective against HPV18 infection than against infection by the other three types targeted by the vaccine, for which a higher percentage of subjects remained seropositive. This observation prompted the proposal that perhaps memory B cells are sufficient to serve as effectors of protection [29]. However, this explanation no longer needs to be invoked, as the apparently lower immunogenicity of the HPV18 VLPs in Gardasil is primarily an artifact of the performance of the serological assay used in the clinical trials. For each of the four HPV types targeted by the vaccine, the assay that Merck used measured the ability of the serum polyclonal antibodies induced by vaccination to compete with a type-specific monoclonal antibody for VLP binding. The binding site of the HPV18 monoclonal antibody they used appears to overlap less consistently with the immunodominant epitopes recognized by the sera of the vaccinees than do the monoclonal antibodies against the other HPV types. Using an alternative “total IgG” assay, 97% of subjects remained seropositive for HPV18 VLPs after four years [30]. As discussed below, it is possible that even the few vaccinees that become seronegative in the most sensitive *in vitro* serologic assays remain protected by circulating antibodies, because very low levels of VLP antibodies appear to be sufficient for protection against infection of cervicovaginal tissue.

In summary, the preponderance of the evidence supports the conclusion that long lived plasma cells (LLPCs) that continuously produce antigen-specific antibodies, and not memory B or T cells, are the key immune effectors that underlie the strong type-restricted protection induced by the HPV vaccines. However, it is important to note that low responders and “breakthrough” infections are rare, and there is no correlation between them, and so the minimum systemic or mucosal antibody level required for protection has not been established yet.

## 3 Immunologic considerations

The exceptionally strong, consistent, and durable antibody responses to the three HPV vaccines is well documented [31]. In healthy young women, seroconversion rates are virtually 100%, peak *in vitro* neutralizing titers of 1000–10,000 are generally obtained, and, after a relatively steep 10-fold drop in titer over the first two years, IgG titers plateau or decline very slowly, stabilizing at levels that are substantially higher than the antibody titers induced by natural infection [32]. Responses in preadolescent girls and boys are even stronger [33], [34]. The stability of antibody responses, now observed for almost a decade [35], [36], is unprecedented for a subunit vaccine.

Surprisingly this pattern of antibody response is observed even after a single dose of vaccine, with stable geometric mean IgG binding and *in vitro* neutralizng titers that are only about 4-fold lower than the plateau titers measured after the standard three doses [8], [37]. Unexpectedly, avidity, as measure in a VLP-based chaotrope ELISA, similarly rose over the first four years after immunization with one or three doses of Cervarix, and then stabilized for both dose regimens [38 and unpublished data].

The long-term antibody levels, regardless of dose number, are almost certainly due to efficient induction of LLPC, which primarily reside in the bone marrow and continuously produce antibodies, probably independent of additional antigen exposure [39]. It is unlikely that successive rounds of memory B cell activation from putative secondary exposure to virion antigens are primarily responsible for the durable levels, as intermittent increases and decreases in antibody levels would be expected if repeated episodic antigen exposure were involved, while the antibody levels in individuals actually remain constant or decrease at a slow rate. In addition, essentially all vaccinees maintain a stable level of antibodies against the VLP types in the vaccine, and it very doubtful that virtually all the women would have experienced immunizing levels of environmental exposure to each of the multiple genital HPV types targeted by the vaccines. Therefore, the central immunological question is why the HPV vaccines are such potent inducers of LLPCs.

The specific structure of the VLPs that comprise the HPV vaccine is probably the key to their ability to efficiently induce LLPCs. The particulate nature and densely ordered repetitive display of B cell epitopes on the surface of the antigen could contribute in multiple ways to LLPC induction. Perhaps most importantly, the ordered display of epitopes at 50–100 Å on the VLP surface is a pathogen-specific danger signal to the humoral immune system [40]. Epitope spacing at this distance is found on the surface of most viruses (HIV being a notable exception [41]), and on other microbial structures, such as bacterial pili.

**Fig. 2:**
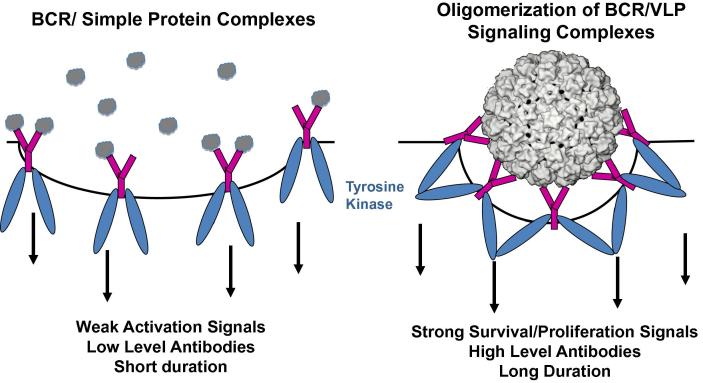
B cell recognition of dense repetitive protein arrays promotes the induction of exceptionally potent and durable antibody responses. BCR = B cell receptors are shown in purple. The BCR-associated tyrosine kinases are depicted in blue. Protein antigens are grey.

Binding and subsequent cross-linking of the B cell receptors (BCR) on the surface of naïve B cells by these ordered repetitive antigens transmit exceptionally strong activation and survival signals [42] (Fig. 2). Naïve B cells generally express both IgM and IgD BCRs. Interestingly, while both monomeric and repetitive antigens can activate IgM BCRs, signaling through IgD is preferentially activated by repetitive antigens, raising the possibility that IgD BCR crosslinking is an important component in the efficient induction of LLPCs by VLPs [43]. It is noteworthy that pentameric subunits of L1 can also induce virion neutralizing antibody responses, however the induced antibody titers are substantially lower than those induced by VLPs (at least when the L1 pentamers have been genetically altered so they cannot self-assemble after injection) [44]. The durability of the antibody responses to assembly deficient L1 pentamers has not been critically evaluated.

Interestingly, high density display of self-antigens on a VLP surface can efficiently break B cell peripheral tolerance and even reactivate anergic self-reactive B cells [45], [46]. The BCRs on a majority of newly produced B cells are thought to bind self-antigens, which renders them functionally anergic [47], [48]. Autoreactive intermediates generated during somatic hypermutation in the B cell follicles on the lymph node may similarly be tolerized. Therefore, there are expected to a rather limited number of immunoglobulin development pathways through which monomeric foreign antigens can generate B cells expressing high avidity antibodies. In contrast, the ability of VLPs to override peripheral tolerance implies that there are a much larger number of developmental pathways available to VLPs than low valency antigens to generate this class of antibodies [41]. The polyvalent interaction of repetitive VLP epitopes might also lead to stable engagement and subsequent B cell activation through BCRs whose affinity, if they were engaged by a monomeric antigen, would be too low to be activating. These conjectures that delineate potential mechanisms for activating a large variety of distinct naïve B cell clones can provide a mechanistic explanation for the remarkable consistency of VLP antibody responses across individuals who have a diverse set of alleles at their immunoglobulin gene loci.

Several additional mechanisms related to the particulate nature of the VLPs may also contribute to their generation of potent antibody responses. First, after parenteral injection, particles of this size (55 nm) readily enter the lymphatic system and traffic to lymph nodes, where they induce primary antibody responses [49]. Second, the closely spaced arrangement of determinants on the VLP surface can lead to the stable binding of natural low avidity IgM and complement, thereby promoting acquisition of the VLPs by follicular dendritic cells, which present antigens for the induction of B cell responses in the lymph node [50]. Third, particles in this size range are efficiently taken up and processed by phagocytic antigen-presenting cells for MHC-II presentation, leading to the induction of potent T helper responses [51]. Fourth, polyvalent binding of the HPV VLPs to human monocytes, macrophages, and dendritic cells induces the release of a variety of cytokines that may promote antibody induction [52].

The above considerations could also help to explain the patterns of antibody responses observed for other classes of vaccines relative to the HPV VLPs. Subunit vaccines composed of monomer or low valency antigens, such as bacterial toxoids and polysaccharide/protein conjugates, consistently induce protective antibody responses only after several doses and require periodic boosting, as the antibody titers continue to wane over time. This is presumably because these antigens do not deliver the strong signals induced by BCR oligomerization that promote differentiation into LLPCs. Interestingly, although the HBV vaccines are multivalent particulate antigens, they behave more like simple subunit vaccines than HPV VLPs in that they often do not induce serocoversion after a single dose and generally fail to induce stable antibody responses [53]. The reason for this difference is not entirely clear. Induction of LLPCs may be limited because the HBV particles are only 22 nm in diameter, the surface antigen spikes in the HBV particles float in a lipid membrane, or because there are a relatively small number of repetitive elements (24 knuckle-like protrusions of the surface antigen for HBV vs 360 L1 molecules arranged into 72 pentamers for HPV) [54]. Each of these factors could limit the potentially critical oligomerization and downstream signaling through the BCRs.

The fact that inactivated virus vaccines are particulate and have a dense array of repetitive surface elements and yet are administered in multiple doses and generally fail to induce stabilizing antibody responses may seem to contradict the hypothesis that these structural elements are the critical features for efficient induction of LLPCs. However, it is likely that the inactivation process (e.g. protein crosslinking with formalin) sufficiently disrupts the dense repetitive array of their surface epitopes to ablate their “virus-like” character [55]. An exception may be the Hepatitis A (HAV) inactivated virus vaccine, which appears to induce durable protective antibody responses after a single dose and therefore may retain a sufficient number of repetitive surface epitopes after inactivation to retain its virus-like character [56]. On the other hand, the observation that live attenuated vaccines, such as yellow fever and vaccinia, induce potent, durable antibody responses and immunity to infection after the primary inoculation in most vaccinees [57] has usually been attributed to the infectious nature of the inoculum. In light of the findings with the HPV vaccines, the alternative explanation, that they are highly immunogenic primarily because they contain authentic virion surface structures, must now be considered.

## 4 Virologic considerations

**Fig. 3:**
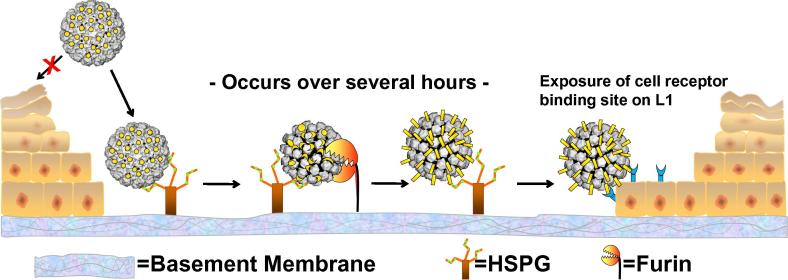
Cervicovaginal HPV infection in a mouse model. A disrupted cerviovaginal epithelium is depicted. “X” indicates the inability of virions to bind the apical surface of intact epithelium. HSPG = heparan sulfate proteoglycan. The L1 capsid structure is depicted in grey. The L2 minor capsid proteins, cleaved by furin protease after a HSPG binding-induced conformational change in the capsid, are shown in yellow.

Papillomaviruses have a unique life cycle in which production of virions occurs only in the terminally differentiated layer of a stratified squamous epithelium. However, completion of its productive life cycle depends upon establishing infection in the cells of the basal layer of the epithelium [58]. To ensure that initial infection occurs only in basal epithelial cells, the virus cloaks its cell surface receptor binding domain until after it has undergone a series of conformational changes. These changes are induced by binding specifically modified forms of heparan sulfate proteoglycans specific to the basement membrane that separates the dermis from the epithelium [59] (Fig. 3).

We have postulated that this unusual strategy of initiating infection on an acellular surface may increase substantially the susceptibility of the virus to serum-derived neutralizing antibodies in two ways [60]. First, exposure of the basement membrane to the virus requires disruption of the epithelial barrier, which results in direct exudation of capillary and interstitial antibodies at these sites. A consequence of this event is that HPV encounters an increasing concentration gradient of systemic antibodies at potential sites of infection. This mechanism can explain why intramuscular injection of the vaccine, a route that is a poor inducer of mucosal antibody responses, can be so effective in preventing a mucosal infection. There is also significant transudation of systemic antibodies via the neonatal Fc receptor in the female genital tract [61]. However, this latter mechanism may play a secondary role in protection, because levels of transudated VLP-specific antibodies in cervical mucus of vaccinated women are 10- to 100-fold lower than serum levels (depending on the stage of the menstrual cycle) [62] and because the vaccines are highly protective against infections of cutaneous epithelia (e.g. external genital warts), which are not routinely bathed in mucus.

The second factor that contributes to increased susceptibility is the exceptional slowness of the initial stages of the papillomavirus life cycle. In a mouse cervicovaginal challenge model, HPV virions remain on the exposed basement membrane for hours before they attach to the epithelial cells that migrate into close the disrupted tissue. Internalization of the cell-bound virus takes several hours, which is also unusually slow [59]. Thus, the virions are exposed to neutralizing antibodies for an exceptionally long time. Relatively high concentrations of passively transferred VLP antisera can prevent infection by inhibiting basement membrane binding. However, lower doses that permit basement membrane binding are nonetheless effective at preventing infection [13]. We speculate that the long exposure of antibody-bound virions on the basement membrane and cell surface makes the complexes highly susceptible to opsinization by phagocytes, which would also be attracted to the sites of trauma [60]. The observation that antibody levels that are more than 100-fold lower than the minimum level detected in the *in vitro* neutralizing assay are able to prevent *in vivo* infection is consistent with the idea that there are potent antibody-mediated mechanisms relevant to *in vivo* inhibition that are not detected *in vitro* [63].

Remarkably low levels of VLP antibodies are protective *in vivo*. For example, in the mouse cervicovaginal model, circulating antibody levels in recipient mice that were 10,000-fold lower than in the donor HPV16 VLP-vaccinated rabbit potently inhibited infection from high-dose HPV16 cervicovaginal pseudovirus challenge [13]. Although the titers of *in vitro* neutralizing antibodies induced by HPV VLP vaccination are approximately 10-fold lower in humans than in rabbits, we nonetheless speculate that the levels of VLPs antibodies in human vaccinees considerably exceed the minimum level required for prevention of genital infection and that protective levels are lower than those that can be reproducibly detected in current *in vitro* antibody binding and neutralizing assays. Therefore, it is our expectation that the fourfold lower, but readily detectable, plateau titers induced by one- versus three-dose vaccine regimens will not substantially reduce the long-term protection induced by the HPV VLP vaccines.
